# Boolean network inference from time series data incorporating prior biological knowledge

**DOI:** 10.1186/1471-2164-13-S6-S9

**Published:** 2012-10-26

**Authors:** Saad Haider, Ranadip Pal

**Affiliations:** 1 Department of Electrical and Computer Engineering, Texas Tech University, Lubbock, 79409, USA

## Abstract

**Background:**

Numerous approaches exist for modeling of genetic regulatory networks (GRNs) but the low sampling rates often employed in biological studies prevents the inference of detailed models from experimental data. In this paper, we analyze the issues involved in estimating a model of a GRN from single cell line time series data with limited time points.

**Results:**

We present an inference approach for a Boolean Network (BN) model of a GRN from limited transcriptomic or proteomic time series data based on prior biological knowledge of connectivity, constraints on attractor structure and robust design. We applied our inference approach to 6 time point transcriptomic data on Human Mammary Epithelial Cell line (HMEC) after application of Epidermal Growth Factor (EGF) and generated a BN with a plausible biological structure satisfying the data. We further defined and applied a similarity measure to compare synthetic BNs and BNs generated through the proposed approach constructed from transitions of various paths of the synthetic BNs. We have also compared the performance of our algorithm with two existing BN inference algorithms.

**Conclusions:**

Through theoretical analysis and simulations, we showed the rarity of arriving at a BN from limited time series data with plausible biological structure using random connectivity and absence of structure in data. The framework when applied to experimental data and data generated from synthetic BNs were able to estimate BNs with high similarity scores. Comparison with existing BN inference algorithms showed the better performance of our proposed algorithm for limited time series data. The proposed framework can also be applied to optimize the connectivity of a GRN from experimental data when the prior biological knowledge on regulators is limited or not unique.

## Introduction

Technological advances in the last two decades have provided numerous approaches to measure various aspects of the regulome in a cell. However, the data generated for specific conditions are still limited both in terms of number of time points and number of samples. Models of genetic regulatory network (GRN) are regularly being inferred from limited time series data on average tissue expression as measured by technologies such as microarray. Selection of a mathematical model to represent a GRN and its inference from limited noisy time series data remains an important problem in systems biology.

The foremost aspect of inference of a mathematical model for explaining a regulatory process is selection of the model. A comprehensive model can provide an accurate picture of the regulation assuming that the parameters of such a model can be correctly inferred. However, we are often faced with limitations on the experimental data which motivates us to design simpler models with the ability to capture the coarse-scale dynamics of the GRN. In this paper, we consider cases where there are only one set of time series transcriptomic or proteomic data generated from a cell line after a specific perturbation. Here, we are considering cell population averaged data as measured by techniques such as microarrays and thus we will start with a deterministic model explaining the average behavior of the system. For a deterministic model, common choices will be Differential Equation (DE) or Boolean Network (BN) type of models. Inference of the parameters of a DE model from minimal data can produce unreliable models as was observed when we tried to infer commonly used linear and non-linear DE models [1] from experimental data of 6 time points (results not shown). A least square cost function was considered and gradient descent was used to optimize the parameters [1]. The inferred DE models were unable to capture any rhythmic behavior present in the experimental data and produced models with significantly different transient and steady-state behaviors for different runs of the parameter optimization procedure. To capture the rhythmic behavior, specific DE models such as Goodwin Models [2] were employed but optimization procedures were unable to infer stable rhythmic models from the 6 data points. The primary reason for the inability of the inferred Goodwin Model to produce a rhythmic behavior was the limited amount of data used for inference. Even though, we interpolated the data, the number of full cycles in the data still remained limited. Thus, we focused on BN types of model. The BN model has yielded insights into the overall behavior of large genetic networks and can be used to model many biologically meaningful phenomena [3,4]. Inference and generation of BNs with specific structure is an open area of research [5].

Our goal in this paper is to provide a BN inference approach from limited time series data and prior biological knowledge on connectivity. The proposed framework can also be applied to optimize the connectivity of a GRN from experimental data when the prior biological knowledge on regulators is limited or not unique. Our analysis will reveal that the chances of generating a BN with small length attractor cycles and satisfying the observed transitions with constraints on connectivity is extremely rare if the regulators of a gene are selected randomly and the data itself lacks structure. We apply our inference approach on time series transcriptomic data of 6 genes and 6 time points from an HMEC cell line following application of epidermal growth factor (EGF) and were able to generate a BN with a biologically plausible singleton attractor structure and satisfying the experimentally observed transitions. The theoretical analysis shows that the generation of such a network from 6 random state transitions and random selection of 3 regulators of every gene is extremely low which in turns suggests that there is structure in the biological data that is exploited by our inference algorithm to arrive at a biologically plausible BN. We next set up an experimental design to compare synthetic BNs with BNs generated through our framework based on state transitions from the synthetic BNs. The results illustrate the capability of the proposed inference technique to generate BNs that are similar to the original BNs by using few state transitions when the connectivity is known.

The paper is organized as follows: The 'methods' section contains (a) a review of BNs and the biologically motivated assumptions and constraints that will be imposed during inference, (b) theoretical analysis of the search space for the inverse problem and (c) Inference Algorithm. The 'results' section contains the results of applying the framework to experimental HMEC data and synthetic BNs; results of comparison with 2 other approaches is also discussed in this section. Further analysis of the results are included in the 'conclusions' section.

## Methods

### GRN model and modeling assumptions

A Boolean network (BN) B = (V, F) on *n *genes is defined by a set of nodes/genes *V *= *x*_1_, ..., *x_n_*, *x_i _*∈ (0, 1), *i *= 1, ..., *n*, and a vector of Boolean functions, *F *= (*f*_1_, ..., *f_n_*), *f_i _*: {0, 1}*^n ^*→ {0, 1}, *i *= 1, ..., *n*. Each node *x_i _*represents the state/expression of the gene *x_i_*, where *x_i _*= 0 means that the gene is OFF and *x_i _*= 1 means that the gene is ON. The function *f_i _*is the predictor function and a subset of the genes *W_i _*⊆ *V *determining the value of the gene *x_i _*at next time step, is called the predictor set for gene *x_i_*.

The biologically motivated assumptions and constraints that we will impose are:

(i) Biological networks usually have sparsity in their connectivity structure. Thus we will restrict our connectivity to *k *where *k *will be typically 3. The initial connectivity structure will be based on prior biological knowledge available from public databases such as *KEGG *(http://www.genome.jp/kegg/), *pathway commons *(http://www.pathwaycommons.org) and *String *(http://string-db.org/). However, the prior biological knowledge is often incomplete to provide an exact connectivity for a gene and thus the available experimental data will be utilized to narrow down the choices.

(ii) Biological networks are usually robust to perturbations and can produce a reproducible trait under changing conditions. The robustness of an inferred model will be measured in terms of coherency of the BN [6] which is the probability that a single gene perturbation of any state in the BN will not alter the basin of attraction of that state. The coherency *ϕ_s _*of an individual state *s *in a BN will be |*s_b_*|/|*s_n_*| where *s_n _*denotes the states that differ from *s *by a hamming distance of 1 and *s_b _*denotes the states among *s_n _*that lie in the same basin of attraction as *s*. The coherency of the BN will be denoted as the mean of the coherencies of the individual states. Among two equally feasible inferred networks, the one with higher coherency will be preferred.

(iii) GRNs usually have small attractor cycles and thus any oscillation observed in our data should be reflected in the Boolean model as a limited state attractor cycle.

(iv) Among two feasible functions, the one with lower inconsistency will be selected. Here, inconsistency refers to same state of the predictor state producing different target output. Let us consider that we have *L *distinct transition from states *S_i _*to *S*_*i*+1 _for *i *= 1, ..., *L*. Each state *S_i _*is a *n *length binary vector *x*_1_(*i*), *x*_2_(*i*), ..., *x_n_*(*i*). Let $y_{i}^{g}\left(0\right)$ and $y_{i}^{g}\left(1\right)$ denotes the number of 0's and 1's respectively observed in the time series data for gene *g *when the decimal value of the state of the *k *length predictor set in the previous time step is *i *where 0 ≤ *i *≤ 2*^k ^*- 1. The measure of inconsistency for gene *g *is $\left(1/L\right)\sum_{i=0}^{2^{k}-1}\text{min}\left(y_{i}^{g}\left(0\right),y_{i}^{g}\left(1\right)\right)$.

### Search space analysis

In this section, we will analyze the size of the search space for the inverse problem of inferring a Boolean model of a GRN from time series data based on connectivity and structural constraints.

Let us consider the case of experimental data of *L *transitions (i.e. *L *+ 1 time points) and *n *genes. The total number of possible BNs from *n *genes is ${\left(2^{n}\right)}^{2^{n}}=N^{N}$where *N *= 2*^n ^*is the number of states. This is equivalent to possible ways of filling a *n *× 2*^n ^*truth table with 1's and 0's. This can be explained through the illustration in table 1 where *n *is assumed to be 3. For example, the value (let's call it *v*_1,1_) of 1st cell in table 1 states that if $X=\left[\begin{array}{c} 0\\ 0\\ 0 \end{array}\right]$ at *time *= *t*, then the value of *x*_1 _is *v*_1,1 _at *time *= *t *+ 1. Since there are 2^3 ^× 3 possible places in the truth table that can be filled with either 1 or 0, the total number of distinct truth tables is $2^{3\times 2^{3}}$.

**Table 1:** Illustration of the number of possible BNs with no constraints on connectivity

*X *→	$\left[\begin{array}{c} 0\\ 0\\ 0 \end{array}\right]$	$\left[\begin{array}{c} 0\\ 0\\ 1 \end{array}\right]$	$\left[\begin{array}{c} 0\\ 1\\ 0 \end{array}\right]$	$\left[\begin{array}{c} 0\\ 1\\ 1 \end{array}\right]$	$\left[\begin{array}{c} 1\\ 0\\ 0 \end{array}\right]$	$\left[\begin{array}{c} 1\\ 0\\ 1 \end{array}\right]$	$\left[\begin{array}{c} 1\\ 1\\ 0 \end{array}\right]$	$\left[\begin{array}{c} 1\\ 1\\ 1 \end{array}\right]$
*x*_1_	0/1	0/1	0/1	0/1	0/1	0/1	0/1	0/1
*x*_2_	0/1	0/1	0/1	0/1	0/1	0/1	0/1	0/1
*x*_3_	0/1	0/1	0/1	0/1	0/1	0/1	0/1	0/1

Here *n *= 3 and $X=\left[\begin{array}{c} {\text{x}}_{1}\\ {\text{x}}_{2}\\ {\text{x}}_{3} \end{array}\right]$ denotes the state of the 3 genes

When we restrict the connectivity to *k*: let's assume that *X *← *R *denotes *X *is regulated by *R*; i.e. $\left[\begin{array}{c} {\text{x}}_{1}\\ {\text{x}}_{2}\\ \begin{array}{c} .\\ . \end{array}\\ {\text{x}}_{n} \end{array}\right]\leftarrow \left[\begin{array}{c} {\text{R}}_{1}\\ {\text{R}}_{2}\\ \begin{array}{c} .\\ . \end{array}\\ {\text{R}}_{n} \end{array}\right]$ where *R_n _*is the regulator set of *x_n _*which has *k *elements. There are $\left(\begin{array}{c} n\\ k \end{array}\right)$ possible ways to select each *R_n_*. So, the total number of possible ways to construct *R *is ${\left(\begin{array}{c} n\\ k \end{array}\right)}^{n}$. For each selection of a regulator set, we have to fill up a truth table of size *n *× 2*^k ^*with 0's or 1's. So the total possible number of BNs is ${\left(\begin{array}{c} n\\ k \end{array}\right)}^{n}\times 2^{n\times 2^{k}}$. For example if *n *= 3 and *k *= 2, table 2 shows that for a specific regulator set R, there are 3 × 2^2 ^cells to fill with 0 or 1 to find a BN. So there are $2^{3\times 2^{2}}$ number of possible BNs for a specific regulator set. As the total number of regulator sets is ${\left(\begin{array}{c} 3\\ 2 \end{array}\right)}^{3}$, the total number of possible BNs is ${\left(\begin{array}{c} 3\\ 2 \end{array}\right)}^{3}\times 2^{3\times 2^{2}}$.

**Table 2:** Illustration of number of possible BNs with constraints on connectivity

*R_n _*→	$\left[\begin{array}{c} 0\\ 0 \end{array}\right]$	$\left[\begin{array}{c} 0\\ 1 \end{array}\right]$	$\left[\begin{array}{c} 1\\ 0 \end{array}\right]$	$\left[\begin{array}{c} 1\\ 1 \end{array}\right]$	
*x*_1_	0/1	0/1	0/1	0/1	←n = 1
*x*_2_	0/1	0/1	0/1	0/1	←n = 2
*x*_3_	0/1	0/1	0/1	0/1	←n = 3

Here *n *= 3 and *k *= 2; *R_n _*denotes the state of 2 corresponding regulator genes

Without restriction on connectivity, knowledge of *L *distinct transitions will fill *nL *places and thus the number of possibilities satisfying the *L *distinct transitions is 2^*n*(*N*-*L*)^. Next, we will consider the case when our connectivity is restricted to *k*. Recall that, in this case, there can be ${\left(\begin{array}{c} n\\ k \end{array}\right)}^{n}$ number of regulator sets each with a truth table of dimension *n *× 2*^k^*. Knowledge of 1st state transition will fill up *n *cells (1 cell in each row) of each truth table. Thus, after 1st transition, there are (*n*2*^k ^*- *n*) unfilled cells in each truth table. Following the first transition, the search space reduces to ${\left(\begin{array}{c} n\\ k \end{array}\right)}^{n}\times 2^{n\times 2^{k}-n}$. Each transition will try to fill up 1 place in each row of a truth table. The probability of hitting an already filled entry in one row on the 2nd transition can be expressed by $\frac{1}{2^{k}}=\frac{1}{m}$ where *m *= 2*^k^*. So, the expected number of entries filled in the second transition is $n\times \left(1-\frac{1}{m}\right)$. To estimate the probability of filling a unique entry on the 3rd transition, we take following approach: Let *P*(*X*) denote the probability to fill a unique position on the 3rd transition; *E*_1 _denote the event that no new entry of a row was filled in the 2nd transition and *E*_2 _denote the event that a new entry of a row was filled in the 2nd transition. Then,

$$P\left(X\right)=P\left(X|E_{1}\right)P\left(E_{1}\right)+P\left(X|E_{2}\right)P\left(E_{2}\right)=\frac{m-1}{m}\times \frac{1}{m}+\frac{m-2}{m}\times \left(1-\frac{1}{m}\right)={\left(1-\frac{1}{m}\right)}^{2}$$

Similarly it can be shown that the probability of hitting a unique place at the 4th transition is ${\left(1-\frac{1}{m}\right)}^{3}$, at the 5th transition is ${\left(1-\frac{1}{m}\right)}^{4}$ and so on.

In general, we can say that the probability of hitting a unique place at the *L*th transition is ${\left(1-\frac{1}{m}\right)}^{L-1}$. This statement can also be proved in another way. There are 2*^k ^*= *m *number of places in each row of a truth table. Let us consider the analogous situation where transition will be considered as putting balls in the places of the truth table and each place can hold more than one ball. To find an empty place at the *L*th transition, previous *L *- 1 balls has to go to (*m *- 1) or less places leaving 1 place definitely empty all the time. There are $\left(\begin{array}{c} m\\ 1 \end{array}\right)$ ways to choose the empty place. And then we can arrange *L *- 1 balls in (*m *- 1) places in (*m *- 1)^*L*-1 ^ways with no constraint on the number of balls in each of (*m *- 1) places. Thus, the total number of ways to put a ball in an empty place on the *L*th transition is $\left(\begin{array}{c} m\\ 1 \end{array}\right)\times {\left(m-1\right)}^{L-1}$. As the total number of ways to put *L *balls in the *m *places is *m^L^*, the probability of filling an empty place on the *L*th transition as $\frac{\left(\begin{array}{c} m\\ 1 \end{array}\right)\times {\left(m-1\right)}^{L-1}}{m^{L}}=\frac{{\left(m-1\right)}^{L-1}}{m^{L-1}}={\left(1-\frac{1}{m}\right)}^{L-1}$.

From *L *distinct transitions, the expected number of distinct places that will be filled in the *n *× 2*^k ^*truth table is $n\times \left(\sum_{i=0}^{L-1}{\left(1-\frac{1}{m}\right)}^{i}\right)=n\times m\left(1-{\left(1-\frac{1}{m}\right)}^{L}\right)=n2^{k}\left(1-{\left(1-2^{-k}\right)}^{L}\right)$. Thus, the expected search space of possibilities following the constraint on connectivity and knowledge of *L *distinct transitions is ${\left(\left(\begin{array}{c} n\\ k \end{array}\right)\right)}^{n}2^{n2^{k}{\left(1-2^{-k}\right)}^{L}}$. The number is still huge, for instance for our biological example presented later *n *= 6, *k *= 3 and *L *= 5, ${\left(\left(\begin{array}{c} n\\ k \end{array}\right)\right)}^{n}2^{n2^{k}{\left(1-2^{-k}\right)}^{L}}\approx 1.65\times 10^{15}$. The size of the search space for the inverse problem remains huge if the connectivity structure is assumed to be unknown.

We next consider the expected number of distinct transitions required to fill up (*m *- 1) places in each row (consisting *m *places) of a truth table with random connectivity. Earlier, we proved that the expected number of distinct places that will be filled from *L *transitions is $n\times m\left(1-{\left(1-\frac{1}{m}\right)}^{L}\right)$. The number approaches *n *× *m *when *L *→ ∞. To get a reasonable idea of the transitions required to almost fill the truth table, we will equate the expression to *n *× (*m *- 1) (i.e. (*m *- 1) in each row). Based on that, our desired $L_{ex}=\frac{\text{log}\left(\frac{1}{m}\right)}{\text{log}\left(\frac{m-1}{m}\right)}$. For *k *= 3 i.e. *m *= 8 the number *L_ex _*equates to 15. In our experimental data, we have *n *= 6 and *k *= 3 which denotes that the expected number of distinct state transitions required to fill up *n *× (2*^k ^*- 1) entries of the truth table will be 15. Unfortunately, we have only 5 distinct transitions in the experimental data and that would require some prior knowledge of the actual connectivity and further constraints to arrive at a plausible BN explaining the data transitions.

Another characteristic of a BN that is desirable from a biological perspective is lack of large length attractor cycles [7]. The ratio of BNs with singleton attractors among all BNs with *N *states is given by (*N *+ 1)^*N*-1^/*N^N ^*[7]. Furthermore, the ratio of BNs with only one singleton attractor among all BNs with *N *states is given by $N^{N-1}/N^{N}=\frac{1}{N}$[7]. Thus, if we randomly generate BNs with 2^6 ^= 64 states, there is a 1 - 1/64 = 0.98 probability that the attractor structure of the BN will not consist of a single attractor. Thus, if our inference approach can produce a BN of 2^6 ^= 64 states with only a singleton attractor, there is a high probability that it is not due to a random event but it might reflect on the use of prior biological connectivity and structure present in the experimental data.

### Inference algorithm

Our propsoed BN inference algorithm is as follows:

*Step 1*: Select *k *regulators for each mRNA/protein. The regulators are selected from prior biological knowledge on connectivity available from public databases. If *k *direct neighbors are not available from the pathway diagram, the remaining ones are selected randomly from the other genes/proteins.

*Step 2*: The entries of the truth table corresponding to the experimental distinct transitions are filled. Here, it is assumed that states of regulators in a single time point determine the state of the next time point of the target gene. In case of inconsistencies, if $y_{i}^{g}\left(0\right)=y_{i}^{g}\left(1\right)$ the value of gene *g *at steady state (i.e. at time point *L *+ 1) is selected. If $y_{i}^{g}\left(0\right)\neq y_{i}^{g}\left(1\right)$, then the state with the maximum value is selected. The remaining unfilled entries are filled with the steady state value of the genes/proteins.

*Step 3*: The score of the generated BN is measured and the number of inconsistencies calculated. The score measures the number of observed transitions in the experimental data reflected in the generated BN. The method of calculating the score is presented in algorithm 1.

*Step 4*: If the score is significantly lower than the maximum possible score (*L(L+1)/2*) and the number of inconsistencies is higher, return to *Step 1 *to change the regulators that were undetermined from the prior pathway knowledge. Run the process for a predefined number of steps and pick the one with the highest score and minimum inconsistency.

**Algorithm 1 **Algorithm for Calculating the Score of a BN

   *Score *= 0

   *Experimental Transitions*: *S*_1 _→ *S*_2 _... → *S*_*L*+1_

   **for ***i *= 1: *L ***do**

      Calculate the transitions in the generated BN from state *S_i _*and remove the ones that are not in the experimental transition data. The truncated transitions in the generated BN will look like $S_{i}\to A_{1}\cdots \to A_{a_{i}}$ where *A_l _*∈ [*S*_+1_, ... , *S*_*L*+1_] for *l *= 1, 2 ... *a_i_*.

      Score = Score + *a_i_*

   **end for**

## Results

**Figure 1:**
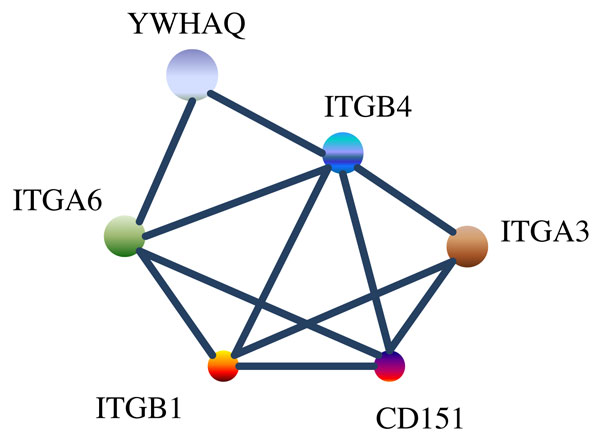
**Pathway of the 6 genes generated from literature search**.

We used transcriptomic and proteomic time series data generated by Rogers *et. al *[8] on the human mammary epithelial cell line (HMEC, strain 184A1) [9] following application of EGF. The transcriptomic data has microarray measurements of 542 genes at 6 time points (1 hr 4 hr 8 hr 13 hr 18 hr 24 hr). The mRNA expressions were binarized using Otsu's method of thresholding [10]. Next, we've searched the literature to locate specific genes involved in the control of mammary cell functions. The initial condition of the original HMEC data suggests that EGFR plays an important role in stimulating the gene/protein expressions. There is evidence in literature [11] stating that EGFR stimulation activates the RNA Binding Protein *CUG *- *BP*1 and increases expression of *C*/*EBPβ - LIP *in Mammary Epithelial Cells. *C*/*EBPβ *is expressed as several distinct protein isoforms (*LAP*1, *LAP*2, and *LIP*). And it is found that *ITGB*4 gene is an activator or interactor of *LAP*2. As we have the data for *ITGB*4 in our *HMEC *dataset, we've checked other genes which are related to *ITGB*4 and also present in the data. We've found 5 genes closely connected to *ITGB*4: *ITGB*1, *ITGA*6, *ITGA*3, *YWHAQ *and *CD*151. After careful observation of the linkages between these six genes from the String database, we've arrived at the connectivity pathway shown in Figure 1. The selection of the connectivity from prior biological knowledge is similar to the approach presented in [12] but the regulatory functions in our approach is found from experimental data.

**Figure 2:**
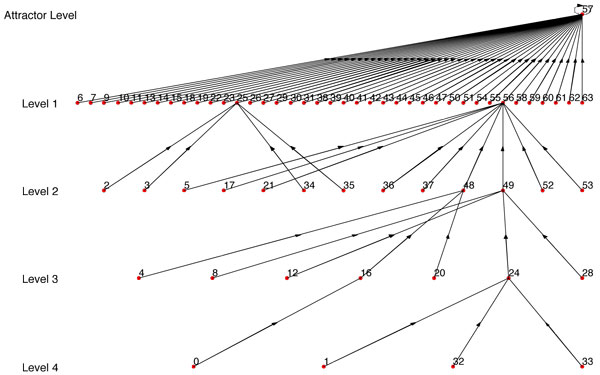
**State transition diagram of the inferred BN**.

**Figure 3:**
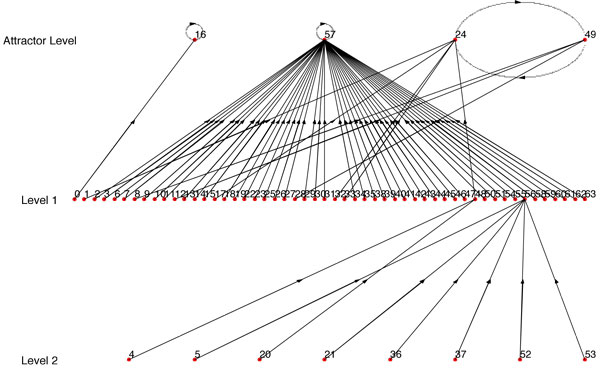
**State transition diagram of the BN inferred using random connectivity**.

For the 6 genes *ITGB*4, *ITGA*6, *ITGA*3, *YWHAQ*, *CD*151 and *ITGB*1, the binarized experimentally observed transcriptomic transitions are 000000 → 000000 → 010000 → 010000 → 111000 → 111001. In decimal representation, the biologically observed transitions are 0 → 0 → 16 → 16 → 56 → 57. The inferred BN using our proposed inference approach and starting with the connectivity structure of Figure 1 is shown in Figure 2. The transitions observed in our inferred network for the states 0, 16, 56, 57 are 0 → 16 → 48 → 56 → 57, 16 → 48 → 56 → 57, 56 → 57 and 57 → 57 producing a score of 11. The maximum possible score considering only distinct transitions is also 11. The inconsistency in the experimental data for the current predictor set is 3 out of 30. The inferred BN has a singleton attractor and no other spurious attractor. As our earlier analysis shows, the number of such structured networks among all BNs of 6 genes is quite low (1/64 = 0.015). Furthermore, the inferred BN has a robust structure with a coherency of 1 as to be expected from a biological network. This shows that our inference algorithm was able to utilize the prior biological knowledge of connectivity and limited experimental data to arrive at a biological plausible robust BN. To show the importance of the prior biological connectivity, we considered a random connectivity structure for gene1 (*ITGB*4) and inferred a BN from the same experimental data which is shown in Figure 3. The BN shown in Figure 3 has a low score of 4, has multiple spurious attractors and has a lower coherency. Thus generation of a robust network with most of the data transitions being reflected in the BN is primarily possible when the correct predictors are selected. Trying out all possible regulatory combinations is computationally expensive as there are ${\left(\left(\begin{array}{c} 6\\ 3 \end{array}\right)\right)}^{6}=64\times 10^{6}$ sets of possible regulator combinations. Use of prior knowledge reduces the search space tremendously.

### Validation with synthetic network models

In the previous section, we showed the result of our inference approach when applied to experimental data. Since the true structure of the Boolean Network for the *ITGB*4 network is not known, we could not exactly compare our results to the original network generating the data. The validation of the generated network was based on the low probability of arriving at a robust structured BN explaining the observed transitions if random connectivity is assumed and there is no biological structure in the experimental data. In this section, we use synthetic BNs to generate data for our inference algorithm and compare the inferred BN with the actual synthetic BN. We took an existing BN (*BN*_1_) and used a path of this BN as our synthetic data (it's the experimental data in our inference algorithm) and applied steps 1 to 3 (inference algorithm) to create a new BN (*BN*_2_) to compare the similarities with *BN*_1_. For step 1, we've used the regulator set of *BN*_1 _(which is known) as our regulator set for the synthetic data. We defined a new similarity measure to compare two BNs that is shown in algorithm 2. For comparison, we have to locate all individual paths in *BN*_1 _which starts with a distinct state and ends with an attractor. The ratio of similarity score (similarity ratio, *R*) and maximum similarity score is 1 if *BN*_2 _perfectly matches with *BN*_1_. It should be less than 1 for mismatch.

**Algorithm 2 **Algorithm for Calculating Similarity Measure of Two Different BNs

   *SimilarityScore *= 0

   *MaxSimilarityScore *= 0

   *NumPath *= Total Number of Paths in *BN*_1_

   **for **i = 1:NumPath **do**

      *L *= Number of Transition in Path(i)

      *Path*(*i*): *S*_1 _→ *S*_2_..→ *S*_*L*+1_

      *Score*(*i*) = 0

      **for ***j *= 1: *L ***do**

         Calculate the transitions in the generated *BN*_2 _from state *S_j _*and remove the ones that are not in Path(i). The truncated transitions in the generated *BN*_2 _will look like $S_{j}\to A_{1}\cdots \to A_{a_{j}}$ where *A_l _*∈ [*S*_*j*+1_, ..., *S*_*L*+1_] for *l *= 1, 2 ... *a_j_*.

         Score(i) = Score(i)+ *a_j_*

      **end for**

      *MaxScore*(*i*) = (*L*(*L *+ 1))/2

      *MaxSimilarityScore *= *MaxSimilarityScore *+ *MaxScore*(*i*)

      **if ***Score*(*i*) == *MaxScore*(*i*) **then**

         *SimilarityScore *= *SimilarityScore *+ *Score*(*i*)

      **else**

         *SimilarityScore *= *SimilarityScore *+ 0.25 * *Score*(*i*)

      **end if**

   **end for**

**Figure 4:**
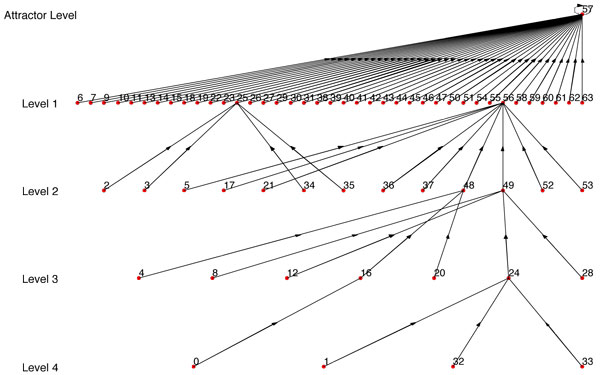
***BN*_2_: an exact match of *BN*_1 _in Fig 2**.

**Figure 5:**
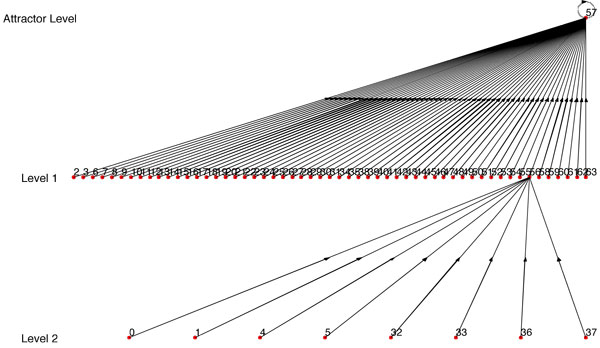
***BN*_2 _has *R *= 0.4214 for synthetic data = 6 → 56 → 57 taken from a path in *BN*_1 _in Fig 2**.

For example, if we take Figure 2 as our *BN*_1 _and one of its path that starts from the bottom most level as synthetic data, then we get *BN*_2 _with *R *= 1. For instance, using synthetic data = 32 → 24 → 49 → 56 → 57, we get Figure 4 as *BN*_2 _which is an exact match of *BN*_1_. If we reduce the number of transitions, then the similarity ratio *R *decreases but we still have some structure in the inferred BN similar to the original network. For instance, if we use synthetic data = 6 → 56 → 57, we get Figure 5 as *BN*_2 _that has a similarity ratio *R *= 0.4214.

If we analyze the structure of Figure 2, we note that it has a singleton attractor and thus a single path of more than four or five transitions is sufficient to reverse engineer the network using the proposed algorithm. Thus, the algorithm fares well for networks with one attractor. However, when we use a network with multitude of attractors, the similarity ratios are much lower if we use a single path of transitions from one basin of attraction. This is quite expected as the algorithm is unable to get an estimate of the other attractors for which we have we no transition data from their basins. Thus for synthetic networks with multiple attractors, we considered transitions from multiple paths of *BN*_1 _as synthetic data and combine them to find the truth table of the Boolean functions. The modifications of our inference algorithm for use of *η *different paths are illustrated next:

*Step 1*: *η *different paths from *BN*_1 _are selected and set as synthetic data (*SD*_1_,*SD*_2_...*SD_η_*). *SD*_1 _will be the path which has greater transition length. If *η*_1 _≤ *η *paths have the same transition length, then the one with singleton attractor is set as *SD*_1_. If all of them have doubleton/singleton attractors, then *SD*_1 _is chosen randomly among those *n*_1 _paths. The other paths are set as *SD*_2_, *SD*_3_....*SD_η _*randomly. Note that the attractors of *SD*_1_,*SD*_2_...*SD_η _*cannot be same. Set of regulators are the same as *BN*_1_.

*Step 2*: The entries of the truth table corresponding to the distinct transitions of *SD*_1_,*SD*_2_...*SD_η _*are filled. Here, it is assumed that the state of regulator in *SD*_1_,*SD*_2 _...*SD_η _*in a single time point determine the state of the next time point of the target gene of *SD*_1_,*SD*_2_...*SD_η _*respectively. If $y_{i}^{g}\left(0\right)\neq y_{i}^{g}\left(1\right)$, then the state with the maximum value is selected. In case of inconsistencies, if $y_{i}^{g}\left(0\right)=y_{i}^{g}\left(1\right)$, the value of gene *g *at steady state of *SD*_1 _is selected. The remaining unfilled entries are filled with the steady state value in *SD*_1_.

*Step 3*: *BN*_2 _is generated based on the truth table and the regulator set. Then *BN*_2 _and *BN*_1 _are compared according to algorithm 2 and similarity score is measured.

**Figure 6:**
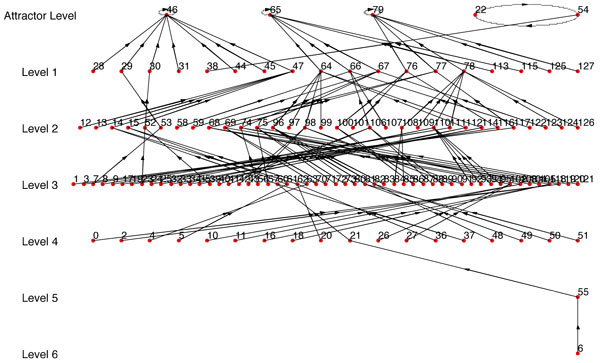
***BN*_1 _with multiple attractors including a doubleton attractor**.

**Figure 7:**
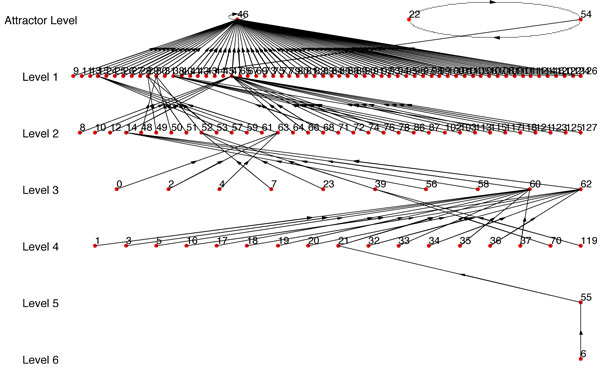
***BN*_2 _where single path is used**. *R *= *R_max _*= 0.1588 as compared to *BN*_1 _in Fig 6.

**Figure 8:**
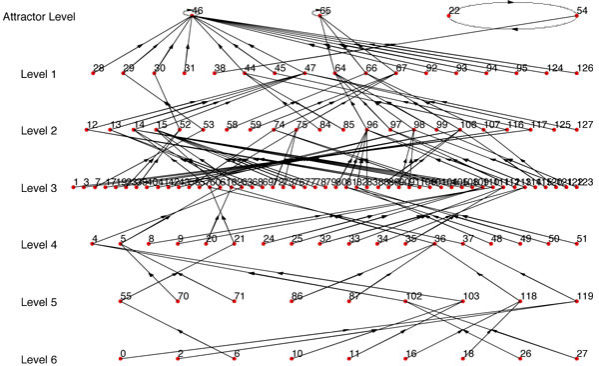
***BN*_2 _where 2 paths are used**. *R *= *R_max _*= 0.5731 as compared to *BN*_1 _in Fig 6.

**Figure 9:**
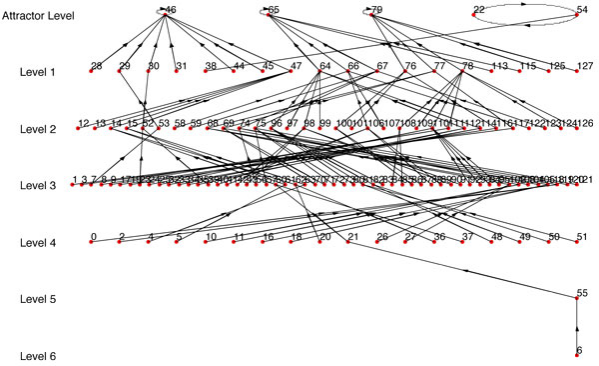
***BN*_2 _where 3 paths are used**. *R *= *R_max _*= 1.0 as compared to *BN*_1 _in Fig 6.

For example, if we use Figure 6 as *BN*_1 _and use one single path of *BN*_1 _as synthetic data, we get *BN*_2 _(Figure 7) with *R *= *R_max_*= 0.1558 (Here *R_max _*refers to the BN with the highest similarity score from the BNs generated using every possible combination of *SD*_1_,*SD*_2_...*SD_n _*that complies with the condition of step 1). But if we use 2 paths as synthetic data, we get *BN*_2 _(Figure 8) with *R *= *R_max _*= 0.5731. We should note that the *BN*_2 _in Figure 7 generated from the single path could capture only 3 of the attractor states (22, 54, 46) and could not capture the other 2 attractor states. Whereas, the inferred network using two paths shown in Figure 8 has 4 of the attractors (22, 54, 46, 65) out of 5 of *BN*_1 _and has a high similarity ratio. As we would expect, combining 3 paths resulted in Figure 9 which is the exact match of Figure 6 with *R *= *R_max _*= 1.0. This implies that the performance of our algorithm increases with the availability of higher number of transitions.

Since *R_max _*is dependent on having the best set of path transitions, we also considered the expected value of *R *when we used at least 4 and 5 transitions for all the paths we are combining. Table 3 contains the summary of the results using Figure 6 as the synthetic BN (*BN*_1_).

**Table 3:** Similarity ratios for BNs inferred from data generated from Fig 6

	*R*_*max*_	*R*_*mean*-4_	*R*_*mean*-5_
1 path	0.1588	0.0904	0.1010
2 paths	0.5731	0.2486	0.3040
3 paths	1.0	0.4243	0.5390

*R*_max _denotes the maximum achieved similarity ratio. *R*_*mean*-*n *_denotes the expected value of the similarity ratio *R *where at least *n *transitions are present in all the paths.

We also considered numerous other BNs with at least 4 attractors as the *BN*_1 _to generate the synthetic data. The details of the experiment is available in the website http://cvial.ece.ttu.edu/ranadippal/tsbn/. The website also contains the state transition diagrams for maximum similarity ratios for 1 path (*BN*_2*/*1*path*_), 2 paths (*BN*_2*/*2*path*_) and 3 paths (*BN*_2*/*3*path*_) corresponding to each *BN*_1_.

For the results reported in Figures 7, 8, 9, table 3 and the website, the regulator structure used for inference was the same as the original BN (*BN*_1_). The gradual increase of values of *R_max _*and *R_mean _*with additional transitions from different paths indicates the reliability of our algorithm. If we have prior biological knowledge on the connectivity of the network with even complicated attractor structures, we can infer a network very similar to the original one with state transitions data from around 2-3 different paths. To further illustrate the importance of prior biological knowledge of connectivity on the success of the inference algorithm, we've randomly changed the predictor set of the BN (*BN*_1_) in Figure 6 and observed the corresponding values of *R_max _*and *R_mean-n _*in table 4 (figures are not shown). We note that the similarity ratios are significantly lower as compared to the values of table 3.

**Table 4:** Similarity ratios for BNs inferred from data generated from Fig 6 with random predictor set

	*R*_*max*_	*R*_*mean*-4_	*R*_*mean*-5_
1 path	0.1132	0.0635	0.0678
2 paths	0.1643	0.0662	0.0681
3 paths	0.2570	0.1088	0.1129

### Comparison with existing BN inference approaches

We compared the performance of our proposed algorithm with (a) Liang *et al. *REVEAL [13] and (b) Zhao *et al. *MDL approach [14].

#### Comparison with REVEAL

REVEAL is a well-known reverse engineering algorithm for inference of genetic regulatory architectures proposed by Liang *et al. *[13]. The REVEAL algorithm receives time series data of *n *genes as input and returns possible regulating genes for each gene along with the regulation function. The set of the regulating genes is a subset of *n *genes.

**Figure 10:**
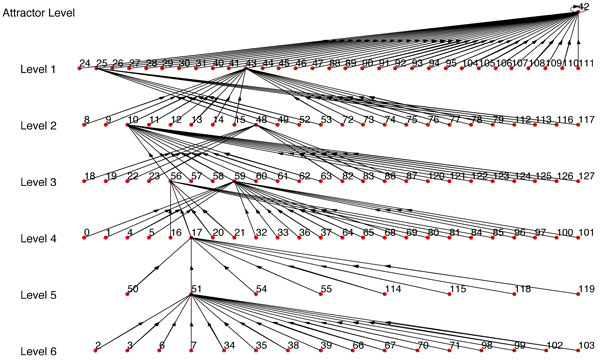
***BN*_2 _inferred using REVEAL**. *R *= *R_reveal _*= 0.0035 as compared to *BN*_1 _in Fig 6.

We've implemented REVEAL algorithm in MATLAB. For convenience of comparison between our approach and REVEAL, we've used synthetic BN where the original regulators and functions are known. We've used our algorithm to infer the BN with maximum similarity ratio (*R *= *R_max_*). Here, each path of the synthetic network was taken as a time series data for our algorithm; and the BN inferred by our algorithm using the same connectivity as the synthetic network was compared with the original one and similarity ratio between these two networks calculated. The path which results in the highest similarity ratio was used as the input time series data for REVEAL algorithm. The BN found from REVEAL was then compared against the original synthetic network and also similarity ratio (*R_reveal_*) was calculated for it. The similarity ratio found by using our algorithm was better than the similarity ratio of the network found by REVEAL. For example, the network *BN*_1 _in Figure 6 was used as synthetic network for generating *BN*_2 _in Figure 7 using our algorithm with *R_max _*= 0.1558; and *BN*_2 _in Figure 10 was found using REVEAL with *R_reveal _*= 0.0035 which is much lower than the *R_max _*= 0.1558. Also, Figure 10 does not have any attractor common with the attractors of the original synthetic BN in Figure 6. We have also conducted comparison with 25 other synthetic networks and the results are reported in the website http://cvial.ece.ttu.edu/ranadippal/tsbn/. The results show that there are single paths from a synthetic BN that will result in high similarity ratios for inferred networks using our proposed algorithm as compared to REVEAL.

#### Comparison with MDL approach

Zhao *et al. *[14] proposed an algorithm for inference of genetic regulatory networks from time series data based on minimum description length (MDL) principle. Applying MDL principle, they generate a set of predecessors (or regulators) for each gene and a conditional probability table for each gene. The conditional probability table contains probabilities of a gene to be 'expressed' (1) and 'not expressed' (0) for a given expression combination of the predecessors.

As we're trying to find a deterministic Boolean network, we've binarized the gene expression based on a conditional probability threshold of 0.5. For example, let's assume that there are 3 genes (*g*_1_, *g*_2 _and *g*_3_) in a network and regulators for *g*_1 _are *g*_2 _and *g*_3_. If the conditional probability *P *(*g*_1 _= 1*|g*_2_*g*_3 _= 00) *>*0.5, we'll take *g*_1 _= 1 if *g*_2_*g*_3 _= 00. Similarly, the value of *g*_1 _for other combination of *g*_2_*g*_3 _is found using the conditional probability table derived by the MDL approach. For the parameter Γ in equation 5 of [14], we used a value of 0.2 which is one of the values used by Zhao *et al. *in their simulations.

**Figure 11:**
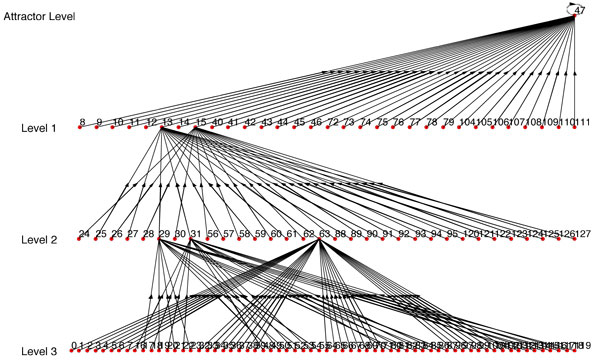
***BN*_2 _inferred using MDL approach**. *R *= *R_mdl _*= 0.012 as compared to *BN*_1 _in Fig 6.

Similar to the comparison technique with REVEAL, we've used the same path from *BN*_1 _in Figure 6 which gave *BN*_2 _with maximum similarity ratio (*R_max_*) (using our approach; Figure 7) as time series data for MDL approach. MDL approach gave the regulators for each gene and the conditional probability table from which the regulation functions are derived using the approach described in previous paragraph. Using the regulator set and the regulation functions, *BN*_2 _in Figure 11 has been inferred with similarity ratio (*R_mdl_*) of 0.012 which is significantly lower than *R_max _*= 0.1558 generated using our approach. None of the attractors of the BN in Figure 11 is common with the attractors of the original synthetic BN in Figure 6. The results of the comparison of our proposed algorithm, REVEAL [13] and MDL approach [14] using several synthetic networks can be found in the website http://cvial.ece.ttu.edu/ranadippal/tsbn/.

Other than the performance with respect to similarity ratio, our approach performs better than both of REVEAL and MDL approaches in elucidating the attractors. Our results also support the claim in Zhao *et al. *[14] regarding the better performance of their algorithm as compared to REVEAL.

## Conclusions

In systems biology, we are often faced with the issue of reverse engineering a GRN model from limited time series data. This article proposes an inference approach utilizing prior biological knowledge of connectivity to generate a BN with biologically plausible state transition structure and explaining the observed transitions in the data. The proposed framework can also be applied to optimize the connectivity of a GRN from experimental data when the prior biological knowledge on regulators is limited or not unique. We validated our algorithm based on experimental data of HMEC cell line and data generated from synthetic BNs with known state transition structure. Through theoretical analysis and simulations, we were able to illustrate that inference of a BN from limited time series data with constraints on connectivity that explains the observed state transitions, is extremely rare if we consider random connectivity. High performance of our proposed algorithm as compared to existing BN inference algorithms that depend on inference of connectivity from the data, further support the advantage of using prior biological knowledge on connectivity. Thus, for cases of limited experimental data, the prior biological knowledge of connectivity should be utilized to arrive at robust BNs with biologically plausible state transition structures. For future research, we will consider combining transcriptomic and proteomic data to reduce the inconsistencies in the data. One of the significant challenges in combined analysis will be the different degradation times for mRNA and proteins.

## List of abbreviations used

BN: Boolean Network; DE: Differential Equation; GRN: Genetic Regulatory Network; EGF: Epidermal growth factor; HMEC: Human Mammary Epithelial Cell; MDL: Minimum Description Length.

## Competing interests

The authors declare that they have no competing interests.

## Authors' contributions

Conceived and Designed the Experiments: SH RP. Performed the Experiments: SH. Analyzed the Results: SH RP. Wrote the article: SH RP. All authors read and approved the final manuscript.
